# The Vasculome of the Mouse Brain

**DOI:** 10.1371/journal.pone.0052665

**Published:** 2012-12-20

**Authors:** Shuzhen Guo, Yiming Zhou, Changhong Xing, Josephine Lok, Angel T. Som, MingMing Ning, Xunming Ji, Eng H. Lo

**Affiliations:** 1 Neuroprotection Research Laboratory, Departments of Radiology and Neurology, Massachusetts General Hospital, Harvard Medical School, Boston, Massachusetts, United States of America; 2 Broad Institute, Massachusetts Institute of Technology and Harvard Medical School, Boston, Massachusetts, United States of America; 3 Department of Pediatrics, Massachusetts General Hospital, Harvard Medical School, Boston, Massachusetts, United States of America; 4 Clinical Proteomics Research Center, Department of Neurology, Massachusetts General Hospital, Harvard Medical School, Boston, Massachusetts, United States of America; 5 Cerebrovascular Research Center, XuanWu Hospital, Capital Medical University, Beijing, Peoples Republic of China; University of South Florida, United States of America

## Abstract

The blood vessel is no longer viewed as passive plumbing for the brain. Increasingly, experimental and clinical findings suggest that cerebral endothelium may possess endocrine and paracrine properties – actively releasing signals into and receiving signals from the neuronal parenchyma. Hence, metabolically perturbed microvessels may contribute to central nervous system (CNS) injury and disease. Furthermore, cerebral endothelium can serve as sensors and integrators of CNS dysfunction, releasing measurable biomarkers into the circulating bloodstream. Here, we define and analyze the concept of a brain vasculome, i.e. a database of gene expression patterns in cerebral endothelium that can be linked to other databases and systems of CNS mediators and markers. Endothelial cells were purified from mouse brain, heart and kidney glomeruli. Total RNA were extracted and profiled on Affymetrix mouse 430 2.0 micro-arrays. Gene expression analysis confirmed that these brain, heart and glomerular preparations were not contaminated by brain cells (astrocytes, oligodendrocytes, or neurons), cardiomyocytes or kidney tubular cells respectively. Comparison of the vasculome between brain, heart and kidney glomeruli showed that endothelial gene expression patterns were highly organ-dependent. Analysis of the brain vasculome demonstrated that many functionally active networks were present, including cell adhesion, transporter activity, plasma membrane, leukocyte transmigration, Wnt signaling pathways and angiogenesis. Analysis of representative genome-wide-association-studies showed that genes linked with Alzheimer’s disease, Parkinson’s disease and stroke were detected in the brain vasculome. Finally, comparison of our mouse brain vasculome with representative plasma protein databases demonstrated significant overlap, suggesting that the vasculome may be an important source of circulating signals in blood. Perturbations in cerebral endothelial function may profoundly affect CNS homeostasis. Mapping and dissecting the vasculome of the brain in health and disease may provide a novel database for investigating disease mechanisms, assessing therapeutic targets and exploring new biomarkers for the CNS.

## Introduction

In recent years, mechanistic investigations into brain function and disease have shifted away from a purely “neurocentric” focus into a more integrative perspective that involves all cell types in the central nervous system [1], [2], [3], [4]. For example, interactions between neurons and glia are required for normal neurotransmission as well as remodeling and recovery after brain injury [5]. Signals from astrocytes and pericytes provide important regulatory mechanisms for the blood-brain barrier [6], [7], [8]. And overlapping mediators underlie crosstalk between neurogenesis, angiogenesis, and common patterning of neural and blood vessel architectures [9].

Blood vessels in the brain are no longer viewed as passive or inert plumbing to simply carry blood flow for oxygen and glucose delivery. Increasingly, it is now recognized that the cerebral endothelium provide a rich source of signaling and trophic factors that influence brain function. Cerebral endothelium can produce growth factors that promote neurogenesis [10]. Cerebral endothelium can release neuroprotective factors such as brain derived neurotrophic factor (BDNF) and fibroblast growth factor (FGF) that defend neurons against a wide range of metabolic and toxic insults [11], [12], [13], [14]. Conversely, “sick” endothelium can contribute to CNS disease. In diabetes, oxidatively stressed cerebral endothelium produce lower levels of neurotrophic factors that may lead to increased neuronal susceptibility to stroke and neurodegeneration [15]. Dysfunctional microvessels and disrupted blood-brain barrier function have been proposed to worsen neuronal dysfunction in Alzheimer’s disease and amyotrophic lateral sclerosis [16], [17], [18]. Hence, understanding the full functional profile of cerebral endothelium may be extremely important for investigations into CNS physiology and pathophysiology.

To date, many studies of brain endothelial gene expression have been performed. The majority of these efforts primarily focus on the blood-brain barrier [19], [20], [21], some of them are from the microvascular fragments [22]. However, a broader approach that connects the entire vascular blueprint to brain function and disease has not been attempted. Here, we propose the concept of a brain vasculome, i.e. a systematic mapping of transcriptome profiles of endothelial cells from brain in comparison with those from two other major organs, the heart and kidney glomeruli, in order to potentially reveal differential vascular function at a whole genome level. Our database here is then dissected to assess the hypothesis that the brain vasculome may contribute to CNS disease in terms of mechanisms and circulating biomarkers.

## Results

### Quality Control of Vasculome Gene Expression

Two levels of quality control were assessed. First, quality and integrity of RNA samples were tested with standard NanoDrop and Bioanalyzer approaches to ensure sufficient RNA concentrations, 260/280 ratios, 28 s/18 s ratios and RNA integrity number (RIN) scores. Second, the quality of microarray hybridization was also assessed by manually checking the distribution of hybridization signals, percentage of positive signals, ratio of 3′ to 5′ end of housekeeping genes (GAPDH and β-Actin), and applying principal component analysis to identify potential outliers. All samples passed these various checkpoints. For the final data sets, RNA from n = 5 mice were pooled per microarray, and n = 3 independent microarrays were used for each group (see Methods).

Next, we asked whether our vasculome was contaminated by parenchymal cells. For the brain vasculome, we compared our data with gene markers for different brain cells in public GEO GSE13379 datasets [23], [24], that contain gene expression profiles for neurons, astrocytes and oligodendrocytes. This analysis demonstrated that genes known to be representative of neurons, astrocytes and oligodendrocytes had extremely low expression levels (signals <50,) in our brain vasculome, whereas gene markers of endothelial cells had much higher expression levels than others (Table 1). These data suggest that our brain vasculome is endothelial-specific and not contaminated by surrounding parenchymal brain cells. Another check of endothelial purity was performed using RT-PCR to assess the expression of gene markers in the vasculome from brain, heart and kidney glomeruli (Table S1). Compared to corresponding whole organ tissue, each vasculome had higher expression of endothelial markers (≥ 3 fold), whereas gene expression levels were enriched for neuron and astrocyte markers in whole brain samples; myocyte markers in heart tissue samples; and kidney tubular markers in kidney tissue samples, respectively (Figure S1). Taken together, these analyzes suggest that the various organ vasculomes were not overtly contaminated with parenchymal genes.

**Table 1:** Differential expression of cell-type specific markers in brain vasculome versus other cell types in brain.

Cell Type	Symbol	Probe ID	Brain vasculome	Astrocyte	Neuron	Oligodendrocyte
astrocyte	2900052N01Rik	1436231_at	3.3209	12.1724	5.5117	5.5370
astrocyte	Acsbg1	1422428_at	3.8742	10.2979	7.1814	6.9079
astrocyte	Gfap	1440142_s_at	2.9647	12.4009	7.2991	7.2095
astrocyte	Gjb6	1448397_at	3.3301	12.3716	8.2681	8.2473
astrocyte	Slc39a12	1436611_at	2.9493	12.4861	8.6637	8.8039
astrocyte	Ttpa	1427284_a_at	3.5321	9.9466	4.9249	4.2301
Neuron	Crh	1457984_at	4.0607	9.8493	11.7976	10.5446
Neuron	Hs3st2	1438624_x_at	3.2656	6.0426	10.2390	8.5537
Neuron	Htr2c	1435513_at	3.4612	6.2973	10.2024	8.5207
Neuron	Mal2	1427042_at	3.6787	9.1258	11.7147	10.1006
Neuron	Necab1	1437156_at	2.6924	7.0119	10.7180	8.6910
oligodendrocyte	Cldn11	1416003_at	5.4823	7.4864	8.6126	12.2323
oligodendrocyte	Ermn	1436578_at	3.7956	5.3308	6.6879	11.9959
oligodendrocyte	Ermn	1440902_at	2.4515	8.0862	9.0134	13.4799
oligodendrocyte	Mag	1460219_at	3.4820	4.6254	8.2041	10.7587
oligodendrocyte	Opalin	1435854_at	4.5864	6.0679	6.8508	12.1927
oligodendrocyte	Pdgfra	1421917_at	4.8939	5.8151	8.1136	10.0721
oligodendrocyte	S1pr5	1449365_at	4.3481	6.1865	8.1488	12.0642
oligodendrocyte	Sox10	1451689_a_at	4.2181	6.0325	7.4529	10.7882
oligodendrocyte	Tmem125	1434094_at	3.4232	3.7906	5.6062	11.1095
oligodendrocyte	Ugt8a	1419063_at	4.8300	5.4295	9.0736	12.6444
endothelial	Cdh5	1422047_at	10.2706	2.1884	2.1632	2.1702
endothelial	Cdh5	1433956_at	8.5466	2.1817	2.1886	2.1702
endothelial	Cldn5	1417839_at	11.6755	3.2007	4.8258	3.6992
endothelial	Flt1	1419300_at	10.3308	2.1824	2.6425	2.1702
endothelial	Flt1	1440926_at	9.9110	2.2568	2.3708	2.1702
endothelial	Flt1	1451756_at	10.5391	2.1877	2.9728	2.2005
endothelial	Flt1	1454037_a_at	11.6309	2.1818	2.1800	2.1702
endothelial	Nos3	1422622_at	7.3330	5.6658	4.4708	5.0355
endothelial	Ocln	1448873_at	9.9552	2.2325	2.5422	2.1702
endothelial	Pecam1	1421287_a_at	10.0662	2.1817	2.1626	2.1702
endothelial	Tek	1418788_at	11.3776	2.2534	2.7882	2.2962
endothelial	Vwf	1435386_at	10.7795	2.7833	3.9346	4.0555

Note: Numbers for log2 signal intensity. Except for brain vasculome, all other data are listed from GSE13379 of GEO (Doyle JP et al. 2008 and Dougherty JD et al. 2010). Brain vasculome represents mean value of 3 samples, astrocyte represents mean value of 6 samples, neuron represents mean value of 23 samples, oligodendrocyte represents mean value of 4 samples. Well-established markers for neurons, astrocytes or oligodendrocytes are highly expressed in their corresponding cell types, while all neuronal,. Astrocytic and oligodendroglial genes have extremely low expression levels (or not detectable) in the brain vasculome. In contrast, endothelial markers are highly expressed in the vasculome and show low levels in other types of cells.

### Brain Vasculome Specific Genes and Enriched Pathways

**Figure 1:**
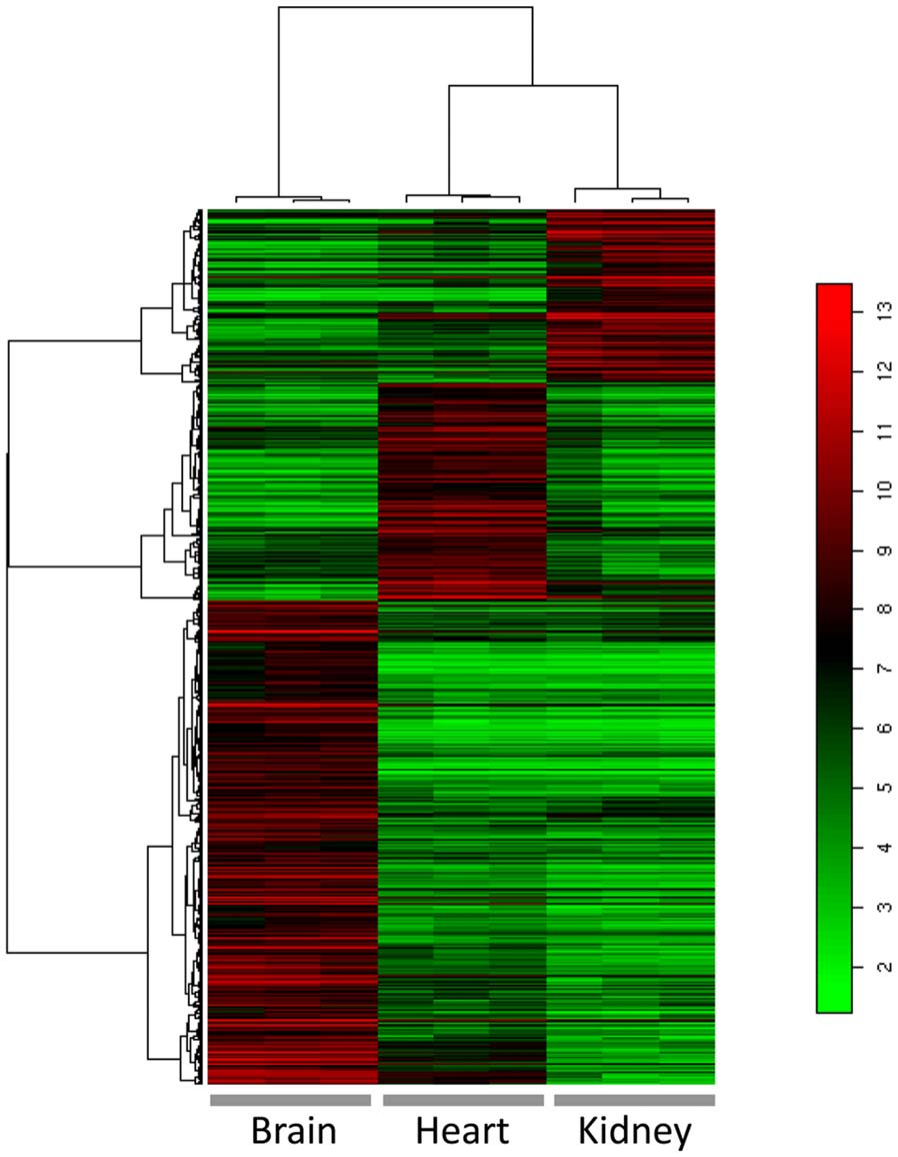
The vasculome of mouse brain is unique and different from those found in mouse heart and kidney. Heatmap for visualization of the expression levels of organ-specific endothelial genes across brain, heart and kidney glomeruli. X-axis represents individual samples and y-axis represents different genes. The expression levels of genes are indexed by color.

Although the microarrays revealed a large amount of data, we only focused on genes whose maximal expression values across all microarrays were greater than 200. Based on these criteria, we identified 3,557 genes expressed in brain endothelial cells. Next, we asked whether this brain vasculome differed from patterns found in our comparative heart and kidney glomerular vasculomes. Applying criteria of p<0.01, fold change ≥ 4, and maximal expression value across all samples >200, we identified 318 probes corresponding to 243 genes found to be highly expressed in brain endothelial cells, 143 probes corresponding to 110 genes highly expressed in heart endothelial cells, and 114 probes corresponding to 81 genes highly expressed in kidney glomerular endothelial cells. A heat-map analysis demonstrated that each vasculome was highly organ-specific. Gene expression patterns in the brain vasculome significantly differed from those in heart or kidney glomeruli (Figure 1).

In the brain vasculome, as expected, blood-brain barrier genes were easily detected. These included occludin (OCLN) and claudin-5 (CLDN5), two major components of tight junctions in the blood-brain barrier. Another known feature of cerebral endothelium is the presence of glutamate receptors that influence barrier function [25], [26], [27]. In our datasets, both ionotrophic and metabtrophic glutamate receptors (Gria2, Gria3, Grin2b and Grm5 for AMPA2, AMPA3, NMDA2B and mGluR5 respectively) were specifically expressed in the vasculome from mouse brain but not heart and kidney glomeruli. Overactivation of glutamate receptors may cause excitotoxicity in neuronal compartments. Similarly, activation of NMDA or mGluR5 receptors could also mediate vascular responses caused by hyperhomocysteinemia and nitrosative stress in brain endothelial cells [28], [29]. The glutamate transporter Slc1a1 (solute carrier family 1, member 1), known as EAAC1/EAAT3, was also enriched in the brain vasculome. In neurons, this transporter plays a key role in regulating synaptic glutamate kinetics. But vascular functions may also exist, since brain endothelial cells co-cultured with astrocytes displayed a polarized brain-to-blood transport of glutamate, suggesting that transporter responses in brain microvessels may participate in the regulation of potentially excitotoxic amino acid concentrations [30]. Overall, the brain vasculome demonstrated many mediators commonly found in neural systems. For example, semaphorins comprise a family of factors that control neurite extension and axon guidance [31], [32]. Here, we saw that Sema3C and Sema4D were enriched in the vasculome of brain compared to heart and kidney. Both Sema3C and Sema4D have been implicated in angiogenesis regulation [33], [34], [35], [36], so their involvement in brain vascular homeostasis may also be important. Finally, another parallel with neural signaling may also be underscored by the detection of CamKIIα (calcium/calmodulin-stimulated protein kinase II alpha) [37], [38], indicating that common responses to intracellular calcium fluxes may occur in both neuronal and non-neuronal systems in the brain.

Using the Fisher’s exact test to probe GO (Gene Ontology) and KEGG databases, many signaling and regulatory pathways were found to be enriched in the brain vasculome (Table 2). These included transporter activity, cell adhesion molecules (CAMs), and the Wnt signaling pathway. Besides well-known brain endothelial transporters such as Glut1 (glucose transporter type 1, or slc2a1, solute carrier family 2, member 1) and P-gp (multidrug resistence poly-glycoprotein, or Abcb1a, ATP-binding cassette, sub-family B, member1A), high levels of transferrin (Tf), transferrin receptor (TfR) and exporter ferroportin (slc40a1) were also detected in the brain vasculome. For cell adhesion molecules, NrCAM (neuronal cell adhesion molecule) appeared to be enriched in the brain vasculome. NrCAM was originally known as a neuron-specific gene required for axon guidance and organization of neural circuitry [39], [40], [41]. However, NrCAM has recently been discovered in dermal and umbilical venous endothelium as well, with potential function in angiogenesis regulation and stress response in endothelial cells [42], [43]. The presence of NrCAM in our initial draft of the brain vasculome but not the heart or glomerular vasculome, further suggest close interactions and potential crosstalk between vascular systems and the organ milieu they inhabit. Similar enrichment in membrane proteins was found in the neurexin network. We detected the expression of neurexin and neuroligin in our mouse brain vasculome. In particular, neurexin-1 showed high level of expression in the brain vasculome rather than heart and kidney vasculomes. Once again, expression of neural-related guidance systems in the brain vasculome suggests crosstalk and signaling functions between blood vessels and brain parenchyma [36], [44].

**Table 2:** Enriched pathways detected in the vasculome of mouse brain.

pvalue	log2 odd ratio	GO term	GO category
1.94E-28	4.34	membrane part	Cellular Component
1.09E-27	4.18	membrane	Cellular Component
8.93E-27	4.24	intrinsic to membrane	Cellular Component
2.21E-25	4.10	integral to membrane	Cellular Component
9.93E-09	5.07	cell junction	Cellular Component
6.22E-15	3.47	transport	Biological Process
7.04E-13	7.41	cell-cell signaling	Biological Process
5.45E-11	4.60	ion transport	Biological Process
1.14E-07	2.42	cell communication	Biological Process
1.63E-05	2.22	anatomical structure development	Biological Process
3.41E-13	4.40	transporter activity	Molecular Function
4.81E-12	4.95	substrate-specific transmembrane transporter activity	Molecular Function
1.71E-11	4.57	transmembrane transporter activity	Molecular Function
3.99E-10	4.60	ion transmembrane transporter activity	Molecular Function
3.90E-05	2.27	signal transducer activity	Molecular Function
**pvalue**	**log2 odd ratio**	**KEGG pathway**	
2.67E-06	7.43	Cell adhesion molecules (CAMs)	
1.57E-03	5.15	Leukocyte transendothelial migration	
3.50E-03	10.95	Alzheimer’s disease	
6.24E-03	3.84	Wnt signaling pathway	
9.72E-03	5.09	Adherens junction	

Note: Analysis based on brain endothelial specific genes in the mouse brain vasculome. These enriched pathways suggest that specific pathways and mechanisms are selectively enhanced in brain compared to heart and kidney glomerular vasculomes.

In addition to physiologic pathways that underlie normal function, pathophysiologic pathways related to inflammation were also expressed in the brain vasculome. In the context of brain injury and neurodegeneration, cytokines and chemokines comprise a key network for regulating inflammation. In this vasculome project, 236 probes were screened for 150 cytokines/chemokines. Overall, low signals were detected for most cytokines/chemokines (<50). Applying the criteria of signal intensity >200, only 17 probes for 11 cytokines/chemokines were expressed in the normal mouse brain vasculome - ccl3, ccl9, ccl27, csf1, cxcl12 (SDF1), kitl, pdgfb, pglyrp1, ptn, socs7 and tgfb2 (Table 3). Compared to heart and kidney glomeruli, ccl3 (chemokine (C-C motif) ligand 3), ccl27 (chemokine (C-C motif) ligand 27) and pglyrp1 peptidoglycan recognition protein 1) appeared to be enriched in the brain vasculome (Table 3). Examination of the existing literature suggested that these may be relevant hits. Ccl3 is released by stimulated brain endothelial cells [45], and it has been reported that it may be elevated in brain vessels of Alzheimer’s disease patients [46]. CCL27 is well known for mediating skin inflammation but has also been detected in the brain [47]. Unlike other chemokines, CCL27 has both secreted and nuclear targeting forms that directly modulate transcription of many response genes, thus any involvement of this factor in the brain vasculome could potentially act as a potent amplifier of inflammation [48], [49]. PGLYRPs (or PGRPs, peptidoglycan recognition proteins) have four isoforms, PGLYRP1-4, that function in antibacterial immunity and inflammation [50]. PGLYRP1 can bind with the key stress response proteins such as Hsp70 and S100A4 to trigger cytotxicity for antibacterial activity [51], [52]. Expression pglyrp1 in the brain have been reported, but its endothelial function is currently unknown [53].

**Table 3:** List of cytokines/chemokines expressed in the vasculome of mouse brain.

				log2 signal intensity		
Probe ID	Entrez ID	symbol	description	brain	heart	glomeruli
1419561_at	20302	Ccl3	chemokine (C-C motif) ligand 3	7.8736	4.9617	3.6407
1430375_a_at	20301	Ccl27	chemokine (C-C motif) ligand 27	8.7402	6.5498	6.4454
1449184_at	21946	Pglyrp1	peptidoglycan recognition protein 1	9.5253	4.9028	3.9403
1448823_at	20315	Cxcl12	chemokine (C-X-C motif) ligand 12	7.6783	7.6424	5.0764
1417936_at	20308	Ccl9	chemokine (C-C motif) ligand 9	7.9595	6.8725	5.3389
1450414_at	18591	Pdgfb	platelet derived growth factor, B polypeptide	8.0818	7.5771	7.3091
1460220_a_at	12977	Csf1	colony stimulating factor 1	8.2981	9.2830	10.8936
1415855_at	17311	Kitl	kit ligand	8.4188	8.2296	7.1033
1426152_a_at	17311	Kitl	kit ligand	8.4408	8.0339	7.2815
1439084_at	20315	Cxcl12	chemokine (C-X-C motif) ligand 12	8.4423	7.7932	4.9062
1415854_at	17311	Kitl	kit ligand	9.0837	9.3912	8.0980
1448117_at	17311	Kitl	kit ligand	9.4871	9.5402	8.4600
1455402_at	192157	Socs7	suppressor of cytokine signaling 7	9.5695	9.2669	9.7154
1450923_at	21808	Tgfb2	transforming growth factor, beta 2	9.6153	7.9357	7.7813
1448254_at	19242	Ptn	pleiotrophin	10.3194	6.4970	9.9998
1417574_at	20315	Cxcl12	chemokine (C-X-C motif) ligand 12	11.9791	11.4499	7.9319
1416211_a_at	19242	Ptn	pleiotrophin	12.3765	7.9964	11.9745

Note: The first three factors (Ccl3, Ccl27, Pglryp1) are enriched in brain versus heart and kidney glomerular vasculomes.

**Figure 2:**
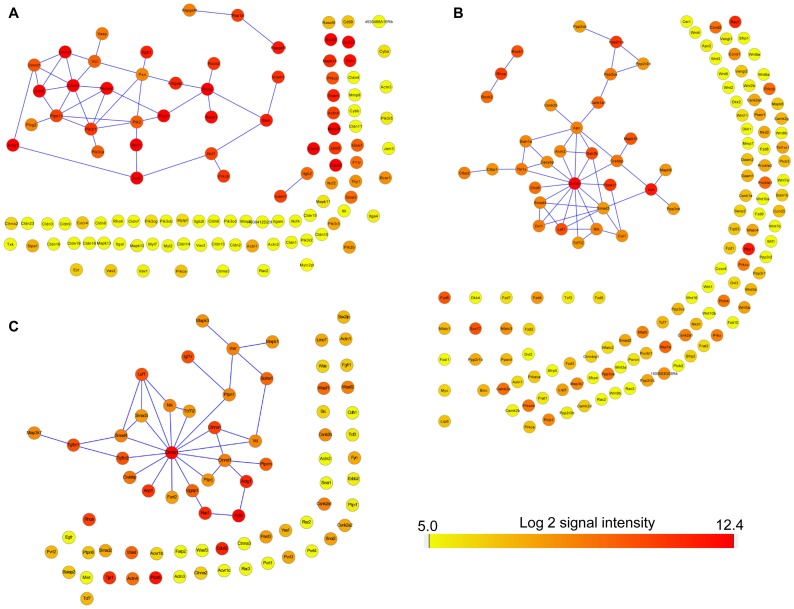
Protein-protein interaction (PPI) networks in the vasculome of mouse brain. **A,** PPI network for leukocyte transendothelial migration. **B,** PPI network for the WNT signaling pathway. **C,** PPI network for adherence junctions. The expression levels of genes in the vasculome of mouse brain are indexed by color.

Another inflammatory example was found in pathways involved in leukocyte transendothelial migration (Figure 2A). The brain vasculome-enriched genes in this pathway included Ncf1 (neutrophil cytosolic factor1, or p47 phox), Prkcb (protein kinase C, beta) and Prkcc (protein kinase C, gamma). Ncf1 is a subunit of NADPH oxidase, a critical enzyme for ROS production in injured or diseased vascular systems [54], [55]. It was reported that Ncf1 mediated the Abeta42 and RAGE ligation induced ROS production and downstream ERK1/2 phosphorylation and cPLA2 (cytosolic phospholipase A2) phosphorylation in cerebral endothelial cells [56]. The PKC family is known to regulate the phosphorylation and uptake of SLC6 family of neurotransmitter transporters [57], also reported to be present in brain endothelial cells and regulate the blood-brain barrier [58], [59], [60]. Whether Prkcb and Prkcc in the brain vasculome contribute to disease phenomena involved in cerebral ischemia, brain injury and neurodegeneration remains to be fully elucidated.

Finally, a prominent network that was enriched in the brain vasculome comprised the Wnt pathway (Figure 2B). Wnt is known to regulate neuronal stem cells, neurogenesis and neuroplasticity [61], [62], [63], [64]. But recently, Wnt signaling has been reported to also participate in the development of CNS vasculature, blood-brain barrier formation, and the protection of endothelial cells after injury [65], [66], [67], [68], [69]. In our draft of the brain vasculome, β-cantenin (CTNNB1) was presented in the hub position of the Wnt protein-protein interaction network, along with brain endothelial-specific genes Axin2, MAPK10 (mitogen-activated protein kinase 10) and Lef1 (lymphoid enhancer binding factor 1). Axin2 is a transcriptional target of active Wnt signaling that also serves to autoregulate and repress the pathway by promoting β-cantenin degradation [70]; In the conditional transgenic mice overexpressing β-Catenin, Axin2 is one of the antagonists changed in the brain [71]. MAPK10 was originally described in neurons but it was recently reported to also mediate endothelial migration via eNOS [72]. And Lef1 is the specific transcriptional factor in the downstream effectors of the Wnt pathway [73], [74]. A critical role of Wnt signaling in cell-cell communication can also be seen because its central hub β-Catenin also serves in the protein-protein interaction network for adherens junctions (Figure 2C) for brain endothelial cells, linking specifically with the brain vasculome genes of Igf1r (insulin-like growth factor 1 receptor), Tgfbr1 (transforming growth factor, beta receptor 1) and Lef1 in this network.

### Angiogenesis and the Brain Vasculome

**Figure 3:**
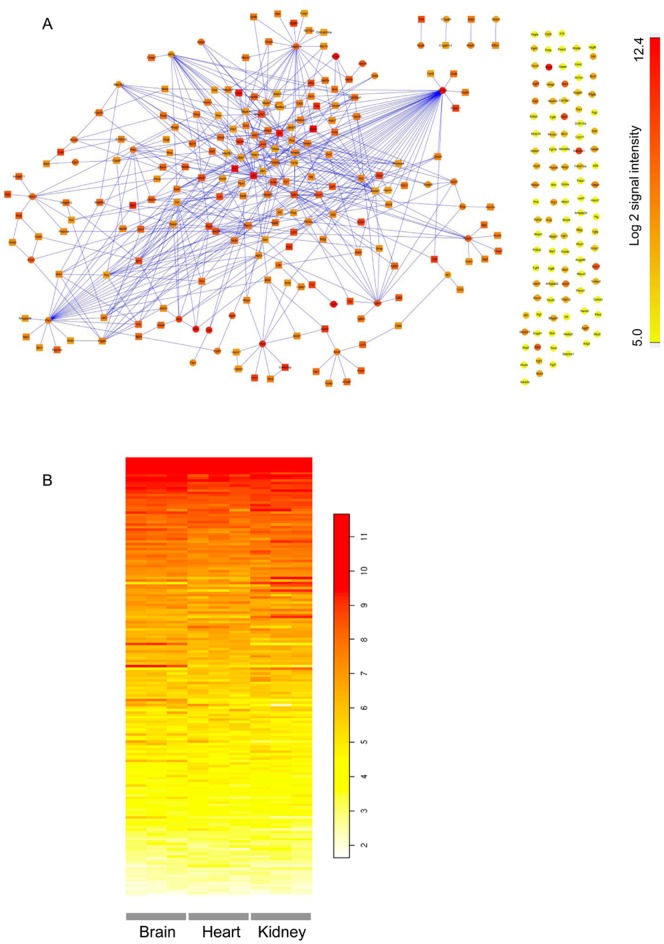
Angiogenesis networks. **A**, Protein-protein interaction network for angiogenesis in the vasculome of mouse brain (including nearest neighbors). Circles for genes in angiogenesis and squares for the neighbor genes. The expression levels of genes in the vasculome of mouse brain are indexed by color. **B**, Heatmap comparison of expression profiles of genes in the VEGF signaling pathway from the vasculome of mouse brain, heart and kidney glomeruli. The expression levels of genes are indexed by color.

In terms of functional networks, angiogenesis should comprise a central part of any vasculome. Probing the GO database revealed a dense protein-protein interaction network for angiogenesis-related genes in the brain vasculome, with hub positions occupied by β-catenin, Rtn4, HIF-1α, Mapk14, Notch1, Ptk2 (protein tyrosine kinase 2, also called focal adhesion kinase 1) and Tgfbr2 (Figure 3A). As described in above, β-catenin is highly expressed in the brain and in the hub positions of other pathways, connecting angiogenesis with these pathways, including Wnt pathway and adherens junctions. Also, Rtn4 (also called Nogo) was highly expressed in the brain. Rtn4 produces 3 isoforms (Nogo-A, Nogo-B, Nogo-C) that may play overlapping roles in vascular as well as neuronal systems in the CNS. Nogo-A is a well-characterized inhibitor of axonal growth and repair [75], whereas Nogo-B is already known to be highly expressed in endothelial cells [76]. Nogo-B regulates vascular homeostasis and remodeling, in part by controlling endothelial cell migration, macrophage infiltration, leukocyte transmigration, and overall inflammation response after tissue ischemia and injury [76], [77], [78]. Overall, Nogo-B may be protective since it is lost after injury [76].

Another angiogeneis gene with high expression levels in the brain vasculome is Gpx-1 (glutathione peroxidase 1), an intracellular antioxidant enzyme that converts hydrogen peroxide to water [79]. Various studies with Gpx-1 transgenic mice suggest that this mediator may be neuroprotective against amyloid toxicity [80], Parkinsons-related pathologies [81], ischemia-reperfusion [82], or trauma [83]. These underlying mechanisms of vascular neuroprotection may broadly include amelioration of cell death, suppression of astrocyte and microglia activation, preservation of BBB function, and a reduction of inflammatory infiltration [82], [84], [85]. Furthermore, Gpx-1 may also contribute to CNS recovery, in part by interacting with hypoxia inducible factor 1 (HIF-1) and its target genes such as VEGF to regulate the angiogenesis process for tissue repair [86]. Gpx-1-deficient mice show decreased recruitment and activation of endothelial progenitor cells after ischemic injury, leading to impaired angiogenesis and revascularization [87].

Within the CNS context, VEGF signaling may be especially important because this mediator may participate in both angiogenesis as well as neurogenesis [88], [89]. Comparison of VEGF signaling pathways showed that these were highly conserved across all three vasculomes in brain, heart and kidney glomeruli (Figure 3B). This may not be surprising since VEGF-mediated angiogenesis may be commonly required network regardless of organ systems. However, it is worth noting that there were two VEGF signaling mediators that appeared to be specifically expressed in the brain vasculome - Prkcb and Prkcc. These two signals were also identified in the leukocyte transendothelial migration network of the brain vasculome (see previous section). Thus, it is possible that particular brain vasculome-specific components may critically influence how the CNS responds to injury and disease. Angiogenesis is a physiological process involving the growth of new blood vessels. This phenomenon is vital not only for organ development but also for tissue repair and wound healing. Insofar as the brain vasculome may be a critical component of CNS plasticity and remodeling, these angiogenesis networks may represent a rich database to probe for potential mechanisms and targets for neurorecovery after stroke, brain injury or neurodegeneration.

### Correlation between Brain Vasculome and CNS Disease Associated Genes

Genome-wide association studies (GWAS) provide valuable information for identifying molecular risk factors and mechanisms for many diseases [90]. For CNS disorders, however, GWAS may be complicated by the fact that disease processes operate not only in neuronal cells but also in other cells from glial and vascular compartments. In the context of stroke and neurodegeneration, pathophysiologic mechanisms are increasingly known to take place in the neurovascular system [1], [2], [3], [4], [16]. So we next asked whether GWAS-defined genes for major CNS diseases could be found in our initial draft of the mouse brain vasculome. Genes implicated in Alzheimer’s disease (AD), Parkinson’s disease (PD) and stroke were compiled from the Database of Genotypes and Phenotypes (dbGaP) at NCBI. A substantial portion of these disease genes was expressed in the brain vasculome –41 AD genes, 53 PD genes and 133 stroke genes (Table 4; complete gene list is provided in Table S2). Representative genes are briefly surveyed below.

**Table 4:** Expression of disease-related genes in the vasculome of mouse brain.

	Alzheimer’s disease	Parkinson’s disease	stroke
GWAS genes in dbGAP	274	364	920
GWAS genes in mouse HomolGene	198	264	643
GWAS genes in mouse M430 2.0	178	239	596
GWAS genes in brain vasculome	41	53	133
p value	0.017	0.016	0.00019
odds ratio	1.50	1.42	1.45

#### Alzheimer’s Disease

CD2-associated protein (CD2AP), as an adapter molecule, is mainly studied in kidney glomeruli. It is highly expressed by podocytes and binds with nephrin to maintain glomerular slit diaphragm function. Mice lacking CD2AP exhibit a congenital nephritic syndrome at early age of 3 weeks [91]. In other tissues, including brain and heart, CD2AP is located in endothelial or epithelial cells, but the functions of CD2AP in brain and heart are still unknown [92].

PAKs (p21-activated kinases), comprising two subfamilies and at least 6 members (PAK1-6), are serine/threonine protein kinases that act downstream of Rho family GTPases Cdc42 and Rac. PAK2 (also known as gamma-PAK), bind with actin and become activated in response to a variety of stresses, and these responses have been implicated in regulation of cytoskeletal structure, apoptosis angiogenesis, vascular integrity and endothelial cell contraction [93], [94], [95], [96]. PAK2 deletion leads to cerebral hemorrhage in redhead zebrafish and this defect is rescued by endothelial-specific expression of PAK2, demonstrating the important role of PAK2 in brain vessels [94].PAK2 may also mediate the VEGF-induced increase of vascular permeability [97]. In the brain, PAK1-3 was reported to regulate the morphology of embryonic cortical neurons, whereas inhibiting Pak activity causing misorientation and branching process of neurons, with increased numbers of nodes, terminals and length of processes [98]. In this regard, PAK may represent yet another example of overlaps between neural and vascular signals in AD pathophysiology.

Ataxin-1 (ATXN1), is a causative gene for spinocerebellar ataxia type 1 (SCA1), with mutation of expanded CAG trinucleotide repeats encoding a polyglutamine tract (polyQ) in the gene [99]. ATXN1 is expressed in both brain and non-neuronal tissues, and may participate in calcium homeostasis, glutamate signaling/excitotoxicity, and Notch signaling pathways [100], [101] through the regulation of transcriptional repression and protein degradation [102], [103], [104]. In primary neuron cultures, knock-down of ATXN1 significantly increases Aβ40 and Aβ42, with increased APP cleavage by β-secretase; while overexpression of ATXN1 decreases Aβ levels [105]. The role of ATXN1 in endothelial cells is not presently well understood, so whether vascular responses in ATXN1 may also affect Aβ homeostasis remains unknown.

Angiomotin (AMOT), first identified as a binding protein to angiostatin, is a transmembrane protein associated with actin. AMOT controls cell migration and motility, cell polarity, tight junction formation and angiogenesis, and also plays critical roles in the tumor suppressor Hippo pathway [106], [107], [108]. AMOT is expressed mostly in endothelial cells and in some epithelial cells, with two protein isoforms, p80 and p130 [109]. The ratio of the two isoforms may regulate the switch between migration and stabilization of endothelial cells [110], [111]. Most of Amot knockout mice die between embryonic day E11 and E11.5 and exhibit severe vascular insufficiency in the intersomitic region as well as dilated vessels in the brain [81]. Whether AMOT contributes to dysfunctional remodeling of brain vessels in the face of progressive Alzheimer’s neurodegeneration is a hypothesis that remains to be fully assessed.

STK24 (sterile20-like kinase 24, also known as Mst3 (Mammalian sterile 20-like kinase-3)) mediates the axon-promoting effects of trophic factors, and may help regulate axon regeneration in damaged neurons [112], [113]. Stk24 has also been reported to regulate cell morphology, migration and apoptosis [114], [115], [116]. In the context of AD or PD, Stk24 may contribute to neuronal Tau phosphorylation, neurite outgrowth and synaptic plasticity modulation by binding with LRRK2 (leucine-rich repeat kinase 2), the most common genetic cause of PD [117]. Recently, Stk24 has also been associated with vascular functions. Stk24, when linked with striatin into a large signaling complex, acts as an essential downstream effector of CCM signaling during cardiovascular development. CCM3 is the disease gene for cerebral cavernous malformations (CCMs), a condition that leads to characteristic changes in brain capillary architecture resulting in neurologic deficits, seizures, or stroke [118]. How these vascular effects interact with neuronal phenomenon remains unclear.

#### Parkinson’s Disease

Regulator of G protein signaling (RGS) proteins form a large family of GTPase-activating proteins (GAP activity) for heterotrimeric G protein alpha subunits that negatively regulate G-protein-coupled receptor signaling. RGS2 selectively accelerates the GTPase activity of Gq/11α and Gi/oα subunits. RGS2 deficiency in mice leads to hypertension and cardiac hypertrophy [119]. Endothelium-specific deletion of RGS2 caused endothelial dysfunction with impaired EDHF-dependent vasodilatation [120]. In the brain, both clinical and animal models showed that lower RGS2 expression is associated with anxiety disorders [121], [122]. In neurons, RGS2 was reported to regulate ionic channel function and synaptic plasticity in the hippocampus [123], [124], [125], [126]. But how RGS2 in brain vessels interacts with neuronal sequelae in PD remains unknown.

HnRNP U (heterogeneous ribonuclear protein U, also scaffold attachment facrot A, SFA) is a multi-functional nuclear matrix protein that has been implicated in multiple inflammatory pathways [127], [128]. Proinflammatory toll-like receptor signaling can stimulate the translocation of hnRNP U from nuclear to cytoplasmic compartments, which then allows it to bind and stabilize mRNA of various proinflammatory cytokines [129]. How these inflammatory actions affect the brain vasculome in PD remains to be determined.

RNF114 (RING finger protein 114, also as ZNF313, zinc finger protein 313), first identified and reported in 2003, is an ubiquitin binding protein and disease susceptibility gene for psoriasis, an immune-mediated skin disorder [130]. RNF114 is reported to regulate a positive feedback loop that enhances pathogenic double-stranded RNA induced production of type 1 interferon by modulating RIG-1/MDA5 signaling [131].

ITSN2 (intersectins 2), a Cdc42 guanine nucleotide exchange factor (GEF), is a multidomain adaptor/scaffold protein involved in clatherin- and caveolin-mediated endocytosis, exocytosis, actin cytoskeleton rearrangement and signal transduction [132]. Several isoforms of ITSN protein can be assembled from alternative splicing, including a brain specific isoform [133]. A role of ITSN-2L in regulating endocytosis within endothelial cells has been reported [134].

PAK1 belongs to the family of p21 activated kinases. In neurons, PAK1 is known to regulate migration [135], [136], spine morphogenesis and synapse formation [137], neuronal polarity [138], and hippocampal long-term potentiation [139]. Besides being a PD GWAS gene, PAK1 may also modulate or bind with other disease proteins, including Fragile X mental retardation 1 (FMR1) for Fragile X syndrome (FXS), the most commonly inherited form of mental retardation and autism [140]; Disrupted-in-Schizophrenia 1 (DISC1) for schizophrenia [141]; ALS2/Alsin for amyotrophic lateral sclerosis (ALS) [142], and Down syndrome cell adhesion molecule (DSCAM) [143]. In endothelial cells, PAK1 may regulate barrier function in different organs [144], [145], and the migration of endothelial cells during angiogenesis [146]. In the context of inflammation, Pak1 is known to assist the invasion of *Escherichia coli* through human brain microvascular endothelial cells [147], [148].

Ubiquitin C-terminal hydrolase 5 (UCHL5), is one of the proteasome 19S regulatory-particle-associated deubiquitinase. Inhibiting the activity of UCHL5 leads to cell apoptosis by altering Bax/Bcl-2 ratios and activating caspase-9 and caspase-3 [149]. Through Rpn13, UCHL5 is recruited in the 26 s proteasome complex during the deubiquitination process. it is reported to regulate the degradation of iNOS and IkappaB-alpha and participated in the process of inflammation and host defense regulation [150], [151]. In the nucleus, UCHL5 is also associated with human Ino80 chromatin-remodeling complex and kept in inactive state, and then activated by transient interaction of the Ino80 complex with proteasome, suggesting that it may cooperate to regulate transcription or DNA repair [152]. UCHL5 interacts with Smads and potentially reverse Smurf-mediated degradation; it also stabilizes type 1 TGF-beta receptor and regulates TFG-beta signaling [153]. It is possible that these inflammatory phenomenons may also be important in the brain vasculome.

TGF-beta signaling is necessary for the development of blood vessels in many organs including brain and heart. Selective deletion of TGF-beta in endothelial cells, but not in neural cells, led to brain-specific vascular pathologies, including intracerebral hemorrhage [154]. Inactivation of TGF-beta type II receptor (Tgfbr2) in endothelial cells in mouse embryo resulted in deficient ventricular separation and haemorrhage from cerebral blood vessels [155]. At the same time, TGF-beta signaling is also important for neural cells. In the midbrain, Tgfbr2 ablation results in ectopic expression of Wnt1/β-Catenin and FGF8, activation of Wnt target genes for regulating neural stem cell expansion [156]. These overlapping actions in neuronal and vascular compartments may allow TGF-beta to play a key role as a PD GWAS gene.

Transcription factor 6 (ATF6) is one of the effectors of endoplasmic reticulum stress [157]. Both oxidized LDL and phospholipolyzed LDL can induce endoplasmic reticulum stress in endothelial cells with ATF6 activation, and this process has been implicated in the initiation of vascular inflammation with progression of atherosclerosis. Via endoplasmic reticulum stress, ATF6 may also regulate responses in angiogenesis and expression of tight junction proteins [158], [159].

#### Stroke

BRM (Brahma), ATPase subunit in the chromatin-remodeling complex SWI/SNF, has important role in gene regulation, inflammation response, tumorigenesis and embryo development and differentiation. BRM is preferentially expressed in brain, liver, fibromuscular stroma and endothelial cell [160]. It is reported that BRM and Brahma/SWI2-related gene 1(Brg1) regulate HIF-1 induced gene expression after hypoxia [161]. Two single nucleotide polymorphisms (SNP) sites were found to be associated with schizophrenia in a Japanese population. A risk allele of a missense polymorphism (rs2296212) induced a lower nuclear localization efficiency of BRM, and risk alleles of intronic polymorphisms (rs3793490) were associated with low SMARCA2 expression levels in the postmortem prefrontal cortex [162].

MBD2 belongs to family of methyl-CpG binding domain (MBD)-containing factors, and mediate epigenetic effects through gene expression regulation. It has been reported that MBD2 was induced in hippocampus within few hours post-ischemia and maintained at high levels for several days [163]. Furthermore, Mbd2 deficient mice were protected against hind-limb ischemia evidenced by improved perfusion recovery and increased capillary and arteriole formation [164]. In vitro experiment also confirmed that knockdown of MBD2 significantly enhanced angiogenesis and provided protection against H2O2-induced apoptosis [164].

NF-κb inhibitor kappa B alpha (NFKBIA, also known as IκB-alpha) is the major regulator of NF-κB activation. Since NF-κB is a central factor in the vast network of inflammation pathways, this stroke GWAS gene is likely to contribute to multiple vascular responses in the brain. In damaged endothelium, dynamic response and inhibitory feedback loops exist between the rapid increase of IκB-alpha and the original NF-κB signal [165]. Links to oxidative stress and vasoregulation may also be important as eNOS-derived nitric oxide can be an endogenous inhibitor of NF-κB activity through IκB-alpha regulation [166].

WNK1, is a member of novel serine/threonine kinase family, With-No-K(lysine), with pleiotropic actions. Intronic deletions in WNK1 gene cause Gordon’s Syndrome, an autosomal dominant, hypertensive and hyperkalemic disorder [167]. WNK1 polymorphisms have also been associated with common essential hypertension [168]. Mechanistically, the WNK1 to ste20/SPAK/OSR1 signaling cascade regulates cation-chloride cotransporters (NKCC1-2), which may be vital for sodium homeostasis regulation, blood pressure response and vascular contractions [168], [169]. Endothelial-specific expression of WNK1 is essential for angiogenesis and heart development in mice, as WNK1 deficiency leads to cardiovascular developmental defects with smaller chambers and reduced myocardial trabeculation, together with defective angiogenesis in both arteries and veins [170]. Overlap with neural responses may also be important. WNK1 mutations have been identified as the cause of hereditary sensory and autonomic neuropathy type II, an early-onset autosomal disease of peripheral sensory nerves. WNK1 can interact with LINGO-1 (a component of tripartite receptor complexes) to regulate nogo-induced inhibition of neurite extension, through activation of RhoA [171].

ADD1 is one of three adducin proteins. ADD1 is a well-known hypertension risk gene. Altered adducin function might cause hypertension through enhanced constitutive tubular sodium reabsorption [172]. Polymorphisms of the ADD1 gene are associated with many physiological responses in hypertensive individuals as well as healthy subjects. For example, the Trp460 ADD1 allele is associated with higher systolic and diastolic blood pressure [173], with increased incidence of peripheral arterial disease (PAD) and coronary heart disease (CHD) [174], increased carotid artery intima-media thickness (IMT) [175], [176], increased risk of stroke [176], and reduced acetylcholine-stimulated forearm blood flow (FBF) response via an impaired endothelium-dependent vasodilation [177]. Again, the study of variants in risk genes suggested that there are physiological interaction between ADD1 and WNK1-NEDD4L pathways to regulate the renal sodium handling, blood pressure and antihypertensive responses to drugs [178]. Furthermore, the overexpression of rat wild type ADD1 in endothelial cells Increased tube formation in vitro and enhanced capillary formation in Matrigel implants in vivo, suggesting ADD1 could regulate angiogenesis process [179].

Among all of these disease genes, there are some with brain vasculome specificity compared to heart and kidney glomeruli. For example, the AD disease gene Pllp (plasma membrane proteolipid, also known as transmembrane 4 superfamily member 11 or plasmolipin), is a myelin structure protein and mainly expressed in brain oligodendrocytes and kidney tubular epithelial cells [180]. It was reported that pllp could form voltage-dependent and K(+)-selective ion channels in the membrane, or act as entry receptor for a kind endogenous retrovirous [181]. The expression of Pllp was signifiacantly reduced in the temporal cortex of patients with schizophrenia and patients with major depressive disorder, suggesting its role in the mental disorders [182], [183]. The PD disease gene Foxf1 (forkhead box F1, also known as HFH-8 or Freac-1), is a developmentally important transcriptional factor. The deficiency of Foxf1 could cause severe abnormalities in the development of many organs including lung, liver and gallbladder, with reduced expression of intergrin-beta3 [184]. As the target of hedgehog, foxf1 and its target gene Bmp4 mediate the induction of vasculogenesis [185] or link hedgehog signaling with Wnt signaling, to regulate the development of organs [186]. The expression of foxf1 in endothelial cells has been reported, and may regulate the inflammation response [187]. For stroke, Apcdd1, Atp2b2, Axin2, ITIH-5 and Slc1a1 are specifically expressed in brain vasculome. As previously discussed, Slc1a1 and Axin2 may be involved in cerebral glutamate handling and vascular development and patterning respectively. Apcdd1(adenomatosis polyposis coli down-regulated 1), a membrane-bound glycoprotein, is the target gene of Wnt/β-Catenin signaling pathway [188], [189], also a novel inhibitor to Wnt signaling in a cell-autonomous manner and acts upstream of β-Catenin [190]. Apcdd1 has an essential role in hair growth [190], or regulate astro-gliogenesis in the brain [191]. ITIH 5 is one of heavy chain subunits of Inter-alpha-trypsin inhibitors (ITIs), a family of serine protease inhibitors. ITIHs stabilize the extracellular matrix (ECM) by interacting with hyaluronic acid, which is a major ECM component [192]. So far, ITIH molecules have been reported to play a particulary important role in inflammation and carcinogenesis [193]. ITIH5 may also be a regulator of human metabolism, as the expression of ITIH5 in adipose tissue was increased in obesity, and associated with measures of body size and metabolism [194]. Hypermethylation in the upstream region of the promoter-associated CpG island of ITIH5, has been detected in breast cancer, and associated with adverse clinical outcome, suggesting ITIH5 as a potential prognostic marker [195]. Atp2b2 is also known as PMCA2 for plasma membrane calcium-transporting ATPase 2, encoding a plasma membrane Ca2+-ATPase type 2 pump, which extrudes calcium from the cytosol into the extracellular space. The mutation of Atp2b2 may cause deafness and imbalance in mice probably by affecting sensory transduction in stereocilia as well as neurotransmitter release from the basolateral membrane [196]. In human primary endothelial cells, Atp2b2 is found to bind with endogenous eNOS, leading to the phosphorylation of eNOS and downregulation of its activity; furthermore, NO production by endothelial cells was significantly reduced by ectopic expression of Atp2b2 [197].

### Overlap between Brain Vasculome and Plasma Protein Databases

By acting as a sensor and integrator of brain dysfunction, endothelial cells within the vast network of cerebral microvessels may represent a critical contributor to CNS biomarkers in circulating blood [198]. We compared our mouse brain vasculome with four independent proteomic databases of human plasma proteins (PMID16041672, PMID16335952, PMID16684767, and PMID18632595) [199], [200], [201], [202], [203], [204]. Protein products corresponding to 754, 1211, 781, and 723 genes respectively, were detected in the mouse brain vasculome (Table 5; complete gene list is provided in Table S3). To be more conservative, we defined a core plasma protein set as the intersection of all 4 databases. This yielded 387 proteins. Out of this core plasma protein dataset, 100 proteins (25.8%) were expressed in the brain vasculome. Whether these “hits” from the normal brain vasculome or future analyses of diseased brain vasculomes may eventually lead to measurable biomarkers in blood remains to be determined.

**Table 5:** Expression of plasma proteins in the vasculome of mouse brain.

Data source	plasma protein	plasma proteins expressed in brain vasculome	%	Reference
PMID:16041672	3365	754	22.4	Muthusamy B. et al, 2005
PMID:16335952	3344	723	21.6	Liu T. et al, 2005
PMID:16684767	2837	781	27.5	Liu T et al, 2006
PMID:18632595	5776	1211	21.0	Qian WJ. et al, 2008
core*	387	100	25.8	

Note: *core is the intersect of all 4 independent data set. Lists of circulating proteins in human plasma were compiled from 4 different proteomic studies, then each study was overlapped with the expression profile of the brain vasculome. A core set of 387 proteins were defined as common proteins detected in all 4 human plasma protein studies. Out of the core set of plasma proteins, 100 proteins were expressed in the brain vasculome.

## Discussion

This study presented initial proof-of-concept for a brain vasculome. The dense network of microvessels in the brain can no longer be simply viewed as inert plumbing. Cerebral endothelium may also be an important source of signaling and trophic factors that communicate with the brain parenchyma. Hence, the brain vasculome may offer a critical tool for investigating how the neurovascular system contributes to the physiology of normal brain function, the pathophysiology of stroke, brain injury and neurodegeneration, as well as provide a database for potential circulating biomarkers that are produced by endothelium in CNS disorders. Our initial analyses suggest that the mouse brain vasculome (1) is unique and significantly different from heart and glomerular vascular systems; (2) is enriched in many vital signaling networks; (3) includes key elements that may contribute to CNS disorders; (4) contain many common genes that have been identified in GWAS databases for stroke, AD and PD; and (5) show significant overlap with plasma protein databases of potential biomarkers in circulating blood.

Taken together, this proof-of-concept study suggests that, when integrated with other genomic and proteomic databases, the brain vasculome may provide a valuable tool for dissecting disease mechanisms, assessing new therapeutic targets as well as searching for new biomarkers in CNS disorders. Nevertheless, there are several caveats that must be kept in mind. First, there is the possibility of gene contributions from non-cerebral-endothelial cell types. Comparisons with other neuronal and glial databases suggest that this may not be a major problem. But we still can not unequivocally exclude this potential source of false positives. Second, although we only focus on endothelial cells in this initial draft of the vasculome, the neurovascular system obviously includes perivascular cells such as pericytes and smooth muscle cells. How the brain vasculome interacts with and is regulated by these other cells warrant deeper studies. Third, our database is based on samples prepared from the entire brain cortex in order to maximize signal-to-noise. But it is likely that the neurovascular system differs in genomic status and function depending on brain region. Whether higher resolution maps of the brain vasculome can be rigorously obtained in the future remains to be determined. Fourth, our vasculome will not operate in isolation but should significantly interact with multiple systems in the entire body. Our data already suggest that vasculome profiles are regulated by the different milieus of each “host” organ. It is likely that the vasculome would also interact with circulating blood cells insofar as genomic signatures in circulating blood are affected by stroke, trauma and various CNS disorders [205]. Fifth, the current draft of our brain vasculome is focused only on mRNA, i.e. the transcriptome. However, other modes of genomic information, including single-nucleotide polymorphism (SNP), copy-number variation (CNV), and epigenomics should also be studied and integrated, in order to obtain a full molecular landscape of the neurovascular system. Ultimately, proteomic and metabolic maps of the brain vasculome should also be extremely useful. Finally, the brain vasculome should be mapped across disease models and states in stroke, brain trauma and neurodegeneration. The normal vasculome presented here only provides a physiologic baseline. Clearly, the vasculome is connected to CNS disease as suggested by the significant overlaps with many GWAS studies of stroke, AD and PD. Mapping the brain vasculome in aged and diseased mouse models may allow us to understand how this system is pathophysiologically affected by and responds to various triggers of injury and disease.

In conclusion, this study provided initial proof-of-concept for a mouse brain vasculome. Mapping and dissecting the full profile of the brain vasculome in health and disease may provide a novel database for investigating disease mechanisms, assessing therapeutic targets and exploring new biomarkers for the CNS.

## Materials and Methods

### Preparation of Microvessel Endothelial Cells

Ten week old male C57BLKS/J mice (Jackson Labs) were used. All experiments were reviewed and approved by a Subcommittee for Research Animal Care of the Massachusetts General Hospital IACUC (Institutional Animal Care and Use Committee) and all these institutionally-approved animal protocols are consistent with the NIH Guide for the Care and Use of Laboratory Animals. To measure the vasculome, we extracted endothelial cells from brain, heart and kidney glomeruli, with modified method from previously published protocols [206], [207]. Briefly, mice were anesthetized by isofluorane and perfused with 8×10^∧^7 inactivated Dynabeads diluted in 40 ml of HBSS (Invitrogen). The cerebral cortex, heart and kidneys were dissected and combined from 5 mice, minced and digested in Collagenase A at 37°C for 30–40 minutes with vigorous shaking (2 mg/ml for cortex and heart, 1 mg/ml for kidney). The digested tissue were mechanically dissociated by titurating, filtered through a 70 µM cell strainer (Becton Dickinson Labware, Bedford, MA), and centrifuged at 500×g for 5 minutes at 4°C. For kidney, materials were further filtered twice with a 100 µM and a 70 µM cell strainer. Cell pellets from brain cortex and heart were resuspended in cold HBSS and mounted on magnetic separator to remove Dynabeads, then supernatant was collected and centrifuged, and incubated with PECAM-1 coated Dynabeads (5 µl for each organ from one mouse) for 30 minutes at 4°C with rotation. A magnetic separator was used to recover bead-bound endothelial cells. Cell pellets from kidney were also resuspended in HBSS and mounted directly on the magnetic separator to select glomeruli containing Dynabeads. Purified glomeruli were further digested with 5 mg/ml of type V Collagenase (Sigma) at 37°C for 30 minutes with agitation, magnetically separated, and then Digested glomeruli were centrifuged, resuspended and incubated with PECAM-1 coated Dynabeads. After washing all materials with HBSS for 5 times, recovered endothelial cells from all organs were lysed with buffer RLT plus for RNA preparation, with RNeasy Micro Plus kit (Qiagen).

### Real Time PCR

Relative expressions of selected markers for different types of cells were tested with RT-PCR, with pre-designed primers and Syber Green system from Bioscience. First strand cDNA was synthesized with QuantiTect reverse transcription system (Qiagen). Date normalization was performed by quantification of the endogenous 18S rRNA, and fold change was measured with 2^−ΔΔCt^ method. The markers for endothelial cells included VE-cadherin, PECAM-1 and eNOS. To ensure that our brain vasculome was not contaminated by parenchymal non-endothelial cells, we also checked markers for astrocytes (Aquaporin-4, GFAP), markers for neurons (MAP-2, Neurogranin), and markers for pericytes and smooth muscle cells (smooth muscle alpha-Actin Acta2), calponin 1 CNN1, desmin, myosin heavy polypeptide 11 Myh11, transgelin Tagln). For heart and glomerular preparations, we checked markers for myocytes (Myh6, NKX 2–5), markers for glomerular podocytes (Nphs-1, Nphs-2) and markers for kidney tubules (Cadherin-16, Claudin-16, Lrp2).

### Transcriptional Profiling with Microarray

Three RNA samples for each organ were individually hybridized to Affymetrix GeneChip Mouse Genome 430 2.0 microarrays, after checking the RNA quantity and quality. RNA concentration was measured by Nanodrop, and the integrity of RNA was tested with RNA integrity number (RIN) score on Agilent Bioanalyzer 2010. All samples were used only when RIN scores were verified to be larger than 7.0. Microarray hybridization and scanning was performed after amplification with the NuGEN Ovation WTA Pico kit and fragmentation and labeling with Encore Biotin Module. Raw expression data for each chip was summarized and normalized using RMA algorithm, to allow direct comparison of results obtained among different chips. The quality of each chip was determined by manually checking mean values, variances and paired scatter plots as well as Principal Component Analysis (*PCA*) *plots.* All chips passed the quality check. Among the large amount of probes/genes, we only focused on genes whose maximal expression values across all microarrays were great than 200, while the probes with intensity less than 200 were eliminated for further analysis.

### Identification of Organ Specifically Expressed Genes

The specific genes between two groups were identified based on both statistical significances, which were determined using SAM algorithm (a variant of t-test and specifically designed for microarray data), and fold change. To minimize false positives, only the genes with maximum expression values across all microarrays greater than 200 were analyzed here. The genes with p<0.01 and fold change >4 were considered as specifically expressed. The combination of p value and fold change threshold serves to eliminate most false positives, as suggested from a large microarray study led by FDA [208]. Fisher’s exact test was used to identify the potential enriched pathways from these brain endothelial specific genes.

### Protein-protein Interaction (PPI) Networks

PPI datasets for human and mouse were downloaded from BIOGRID database at version 3.1.71. Since there were only 2314 proteins and 4118 interactions in the mouse PPI dataset, we transformed human PPI information into mouse’s based on the homolog genes between human and mouse according to NCBI HomolGene database. The human PPI dataset contained 10121 proteins and 52693 interactions. After combining the native mouse dataset and the transformed mouse dataset and deleting repeated records and self-self interaction records, a mouse PPI network with 9189 proteins and 36073 interactions was built. Since not all proteins in the networks are expressed in the endothelial cells under this study, we further shrink the network to EC-specific PPI (EC-PPI) network by deleting the proteins that are not expressed and their corresponding interactions. The EC-PPI contains 4243 proteins and 10825 interactions. The properties of network were calculated with IGRAPH package in *R*. The PPI networks were visualized by Cytoscape software with force-directed layout.

### GWAS and Plasma Protein Databases

Genome-wide- association-studies of disease select the risk genes for the disease. GWAS-identified disease genes for stroke, Alzheimer’s disease and Parkinson’s disease were collected (dbGAP: http://www.ncbi.nlm.nih.gov/projects/gapplusprev/sgap_plus.htm) to analyze the expression of such disease-related genes in endothelial cells. The expression of human plasma proteins were also tested in the brain vasculome. Human plasma proteins determined by proteomics from 4 different studies were used [199], [200], [201], [202], [203], [204]. A core set of human plasma proteins was build with proteins detected in all of these 4 studies, consisting of 387 individual proteins. It is worthwhile to notice that GWAS and plasma protein databases evolve and grow over the time, correlations with our brain vasculome will have to be continually re-assessed in future studies.

### Statistical Methods

All statistical analyses were performed with the statistics software R (Version 2.6.2; available from http://www.r-project.org) and R packages developed by the BioConductor project (available from http://www.bioconductor.org). Overall, raw expression data for each chip was summarized and normalized using RMA algorithm, genes with maximum expression levels across all microarrays great than 200 were considered for further analysis. Organ specifically expressed genes were identified using SAM algorithm; Fisher’s exact test was used to identify the enriched pathways from these organ specific genes. Only genes with p<0.01 and fold change >4 were considered as specifically expressed. The combination of p value and fold change threshold serves to eliminate most false positives, as validated by a large microarray study led by FDA [208]. Fisher’s exact test was also used to test the enrichment of GWAS genes for each disease in the vasculome of mouse brain.

## Supporting Information

Figure S1 **Purity of isolation protocols for brain, heart and kidney glomerular endothelial cells.** The expression of different cell type specific genes were tested by RT-PCR, and compared between endothelial cells and corresponding whole tissue samples.
(PDF)

Table S1 **List of endothelial genes specifically expressed in brain, heart and kidney glomeruli.**
(XLSX)

Table S2 **Full list of GWAS disease associated genes expressed in brain vasculome.** The label “brain EC expressed” indicates whether the gene is expressed in brain vasculome (True) or not (False).
(XLSX)

Table S3 **Full list of plasma proteins expressed in brain vasculome.**
(XLSX)
