# Supplementary figures and images for: The Vasculome of the Mouse Brain
Source: PLoS One. 2012 Dec 20;7(12):e52665. doi: 10.1371/journal.pone.0052665 (PMC3527566; doi:10.1371/journal.pone.0052665)

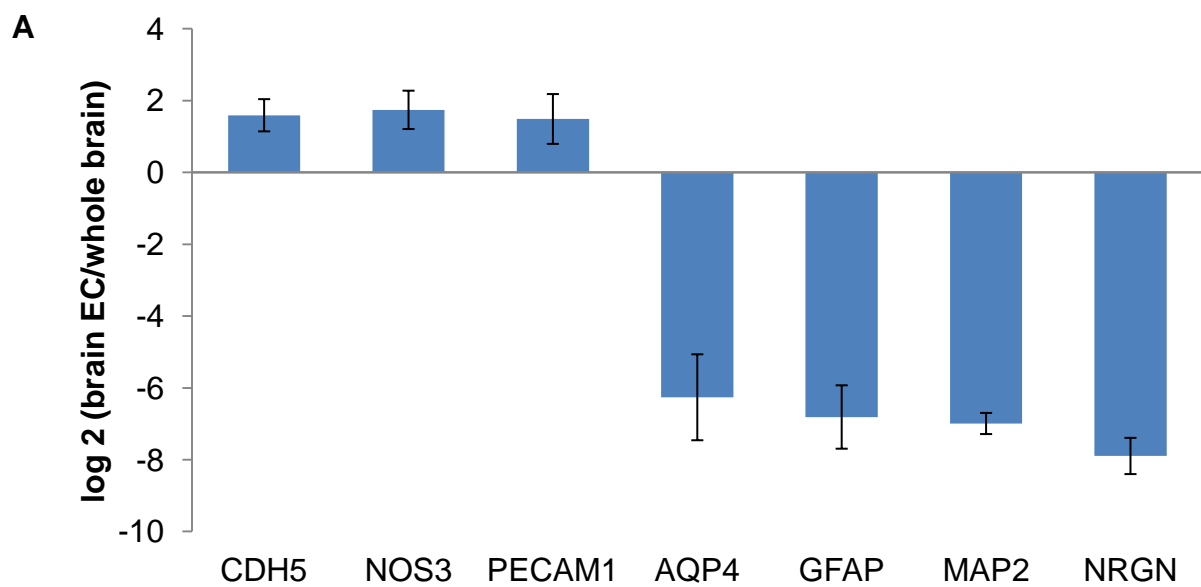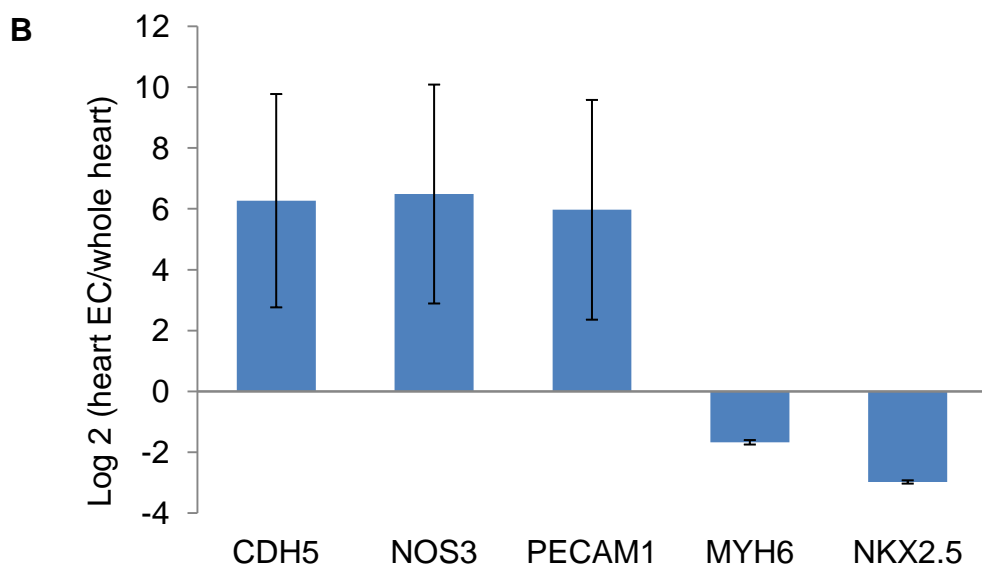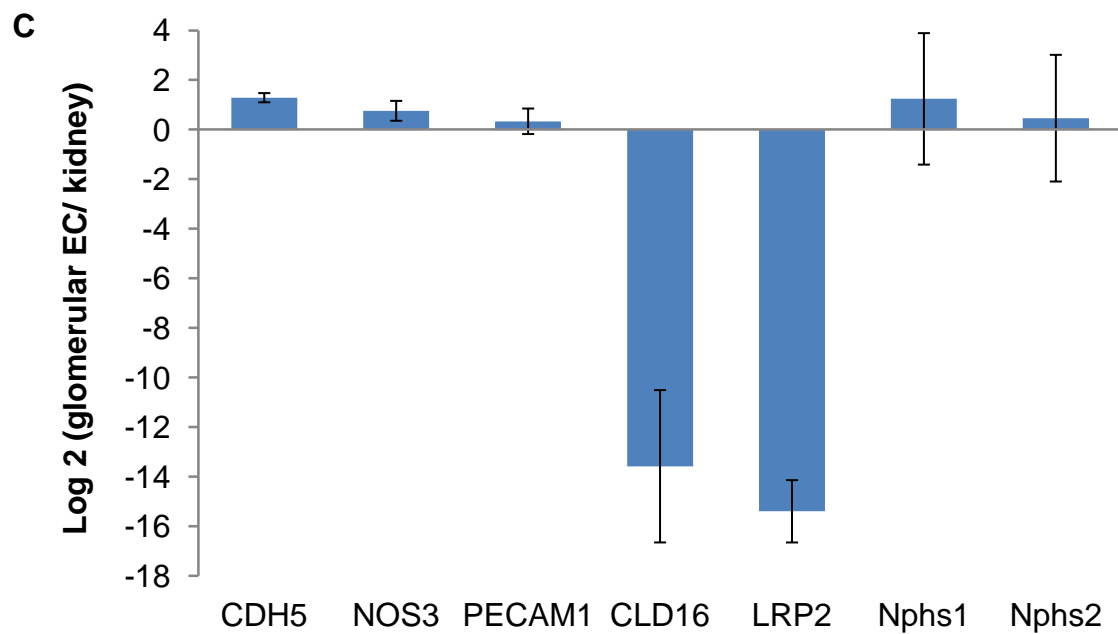

Supplement: Figure S1 — Purity of isolation protocols for brain, heart and kidney glomerular endothelial cells. The expression of different cell type specific genes were tested by RT-PCR, and compared between endothelial cells and corresponding whole tissue samples. (PDF) [file pone.0052665.s001.pdf]
